# Imaging Modalities Employed in Diabetic Retinopathy Screening: A Review and Meta-Analysis

**DOI:** 10.3390/diagnostics11101802

**Published:** 2021-09-29

**Authors:** Piotr Kanclerz, Raimo Tuuminen, Ramin Khoramnia

**Affiliations:** 1 Hygeia Clinic, 80-286 Gdańsk, Poland; 2 Helsinki Retina Research Group, Faculty of Medicine, University of Helsinki, 00014 Helsinki, Finland; raimo.tuuminen@helsinki.fi; 3 Eye Centre, Kymenlaakso Central Hospital, 48100 Kotka, Finland; 4 The David J. Apple International Laboratory for Ocular Pathology, Department of Ophthalmology, University of Heidelberg, 69120 Heidelberg, Germany; Ramin.Khoramnia@med.uni-heidelberg.de

**Keywords:** diabetic retinopathy, fundus photography, mydriatic photography, screening, smartphone-based imaging, ultra-wide-field scanning laser ophthalmoscope, diabetic macular edema

## Abstract

Introduction: Urbanization has caused dramatic changes in lifestyle, and these rapid transitions have led to an increased risk of noncommunicable diseases, such as type 2 diabetes. In terms of cost-effectiveness, screening for diabetic retinopathy is a critical aspect in diabetes management. The aim of this study was to review the imaging modalities employed for retinal examination in diabetic retinopathy screening. Methods: The PubMed and Web of Science databases were the main sources used to investigate the medical literature. An extensive search was performed to identify relevant articles concerning “imaging”, “diabetic retinopathy” and “screening” up to 1 June 2021. Imaging techniques were divided into the following: (i) mydriatic fundus photography, (ii) non-mydriatic fundus photography, (iii) smartphone-based imaging, and (iv) ultrawide-field imaging. A meta-analysis was performed to analyze the performance and technical failure rate of each method. Results: The technical failure rates for mydriatic and non-mydriatic digital fundus photography, smartphone-based and ultrawide-field imaging were 3.4% (95% CI: 2.3–4.6%), 12.1% (95% CI: 5.4–18.7%), 5.3% (95% CI: 1.5–9.0%) and 2.2% (95% CI: 0.3–4.0%), respectively. The rate was significantly different between all analyzed techniques (*p* < 0.001), and the overall failure rate was 6.6% (4.9–8.3%; I^2^ = 97.2%). The publication bias factor for smartphone-based imaging was significantly higher than for mydriatic digital fundus photography and non-mydriatic digital fundus photography (b = −8.61, b = −2.59 and b = −7.03, respectively; *p* < 0.001). Ultrawide-field imaging studies were excluded from the final sensitivity/specificity analysis, as the total number of patients included was too small. Conclusions: Regardless of the type of the device used, retinal photographs should be taken on eyes with dilated pupils, unless contraindicated, as this setting decreases the rate of ungradable images. Smartphone-based and ultrawide-field imaging may become potential alternative methods for optimized DR screening; however, there is not yet enough evidence for these techniques to displace mydriatic fundus photography.

## 1. Introduction

Dramatic changes in lifestyle have led to an increased risk of noncommunicable diseases, such as type 2 diabetes [1]. The prevalence of diabetes mellitus (DM) has been steadily increasing over the past three decades from an estimated 108 million in 1990 to over 415 million people worldwide, or 1 in every 11 adults [2,3,4]. The most prominent increase is noted in low- and middle-income countries.

Diabetic retinopathy (DR) is the leading cause of vision loss both of working-age adults and of preventable blindness globally. In a meta-analysis, Yau et al. estimated that the prevalence of any DR among diabetic subjects might reach 34.6% (95% confidence interval [CI]: 34.5–34.8), while the prevalence of vision-threatening diabetic retinopathy (VTDR) is 10.2% (95% CI: 10.1–10.3) [5]. The risk of DR is higher in individuals with type 1 diabetes, compared to those with type 2 diabetes. Hyperglycemia remains the most important modifiable risk factor for DR [6].

The development of a screening program for DR in Europe was encouraged by the St. Vincent Declaration in 1989 [7], which has set a target to reduce new cases of blindness by one third within a 5-year period. In terms of cost-effectiveness, screening for DR is a critical aspect of DM management [8,9]. Screening for DR is predominantly warranted by the fact that the major complications—macular edema and proliferative DR—respond to treatment [10,11]. According to the International Council of Ophthalmology Guidelines for Diabetic Eye Care 2017, examinations performed for DR screening should involve visual acuity assessment with current spectacles and retinal evaluation (ophthalmoscopy or fundus photography) [12]. In recent years, an important development was noted, particularly in retinal imaging techniques. The aim of this study was to review the imaging modalities employed for retinal examination in diabetic retinopathy screening. The article did not evaluate methods for DR grading, nor deep-learning algorithms for automated DR detection, which may, however, play a significant role in the future [13].

## 2. Materials and Methods

### 2.1. Literature Search

The PubMed and Web of Science databases were the main sources used to investigate medical literature. An extensive search was performed to identify relevant articles concerning “imaging”, “diabetic retinopathy” and “screening” up to 1 June 2021 (Appendix A). The following keywords were used in various combinations: diabetes, diabetic, retinopathy, macular edema, screening, imaging, fundus, photography, and scanning laser ophthalmoscopy. Of the studies retrieved, we reviewed all publications in English and abstracts of non-English publications. The reference lists of the articles analyzed were also considered as a potential source of information. We attempted to present all publications, analyzing the accuracy of various retinal imaging methods employed for DR screening. Emphasis was placed on studies published after the meta-analysis by Bragge et al. [14] and Hu et al. [15]; however, in contrast to those studies, we did not evaluate the performance of different manual methods of the eye examination and aimed to analyze the performance of different technologies. Our study did not aim to compare the accuracy of automated vs. manual analysis, but solely to evaluate the utility of technical methods for obtaining images. Studies were critically reviewed to create an overview and guidance for further research. No attempts were made to discover unpublished data. In addition to the PubMed and Web of Science searches, selected chapters from relevant textbooks were included.

### 2.2. Statistical Analysis

Articles were included in our statistical analysis if they met the following criteria: (i) the study evaluated an imaging modality, and the outcome of interest was the detection of diabetic retinopathy; (ii) the study defined a reference standard for DR detection to which the imaging method was compared; (iii) a threshold for DR detection was defined; and (iv) the sensitivity and specificity for DR detection was specified, or data were given to calculate them. If any investigation presented more than one threshold level for DR detection (e.g., the detection of any DR or alternatively VTDR) the performance for all thresholds was analyzed. In studies comparing the performance of conventional photography and digital photography with the reference standard, we analyzed only outcomes for the digital method. If more than two imaging techniques were applied within the study, all methods were included in the analysis. Meta-analyses were performed by using Stata 14.2 (StataCorp, College Station, TX, USA) environment, i.e., by employing two Stata routines, namely, METAAN (random-effects meta-analysis command) for failure rate calculations and MIDAS (Meta-analytical Integration of Diagnostic Accuracy Studies) for appraisal of sensitivity and specificity of the investigated diagnostic tests. Due to the observed heterogeneity between the studies, random-effects models were applied. When assessing subgroup differences in the meta-analyses, a Chi-squared test was used. The level of *p* < 0.05 was deemed statistically significant.

## 3. Results

The search identified 2137 unique articles. After removing duplicates and irrelevant studies, 148 articles were included in the review. Table 1 presents the testing accuracy in studies on imaging modalities used for detecting diabetic retinopathy.

### 3.1. Technical Failure Rate

**Figure 1:**
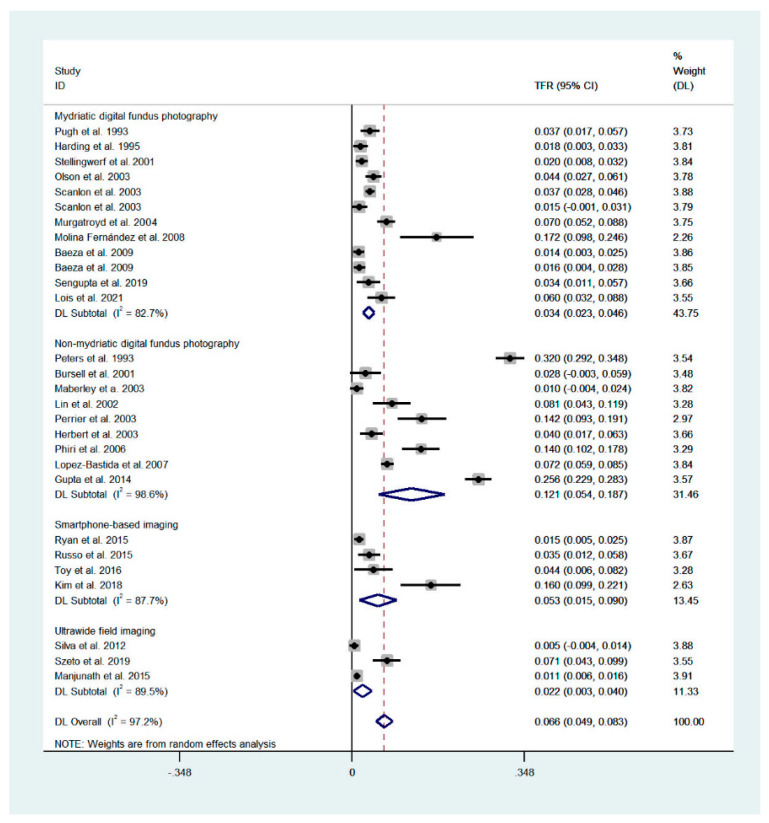
Forest plot presenting the technical failure of the analyzed techniques. Overall values for mydriatic fundus photography: 3.4% (95% CI: 2.3–4.6%, I^2^ = 82.7%), for non-mydriatic fundus imaging: 12.1% (95% CI: 5.4–18.7%, I^2^ = 98.6%), smartphone-based imaging: 5.3% (1.5–9.0%; I^2^ = 87.7%), ultrawide-field imaging: 2.2% (0.3–4.0%; I^2^ = 89.5%). Overall, for all techniques: 6.6% (4.9–8.3%; I^2^ = 97.2%). Weights are calculated from random effects analysis.

The technical failure rates for mydriatic digital fundus photography, non-mydriatic digital fundus photography, smartphone-based imaging and ultrawide-field imaging were 3.4% (95% CI: 2.3–4.6%), 12.1% (95% CI: 5.4–18.7%), 5.3% (95% CI: 1.5–9.0%) and 2.2% (95% CI: 0.3–4.0%), respectively (Figure 1). The failure rate was significantly different between all pairs of the analyzed techniques (*p* < 0.001). The overall failure rate for all techniques was 6.6% (4.9–8.3%; heterogeneity [I^2^] = 97.2%).

### 3.2. Sensitivity and Specificity in Cases without Technical Failure

The articles included in the study are presented in Table 1; ultrawide-field imaging studies were excluded from the final analysis, as the total number of patients included was too small.

**Figure 2:**
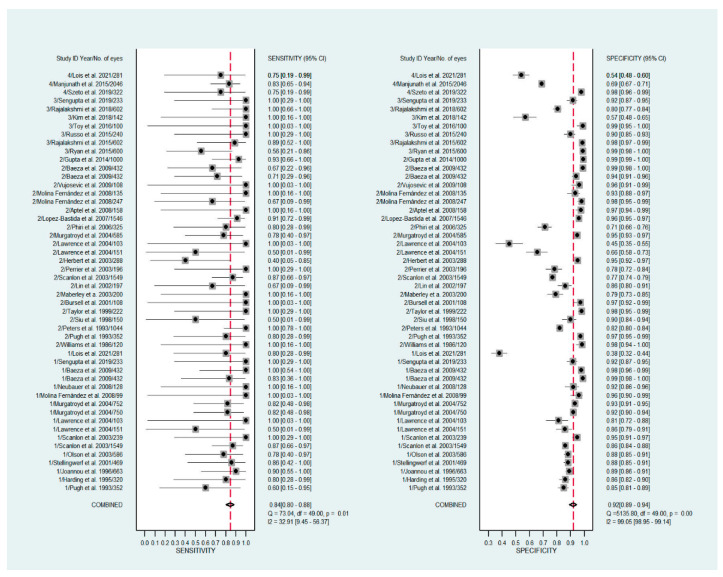
Forest plots for the sensitivity and specificity of mydriatic, non-mydriatic and smartphone-based imaging fundus imaging.

**Figure 3:**
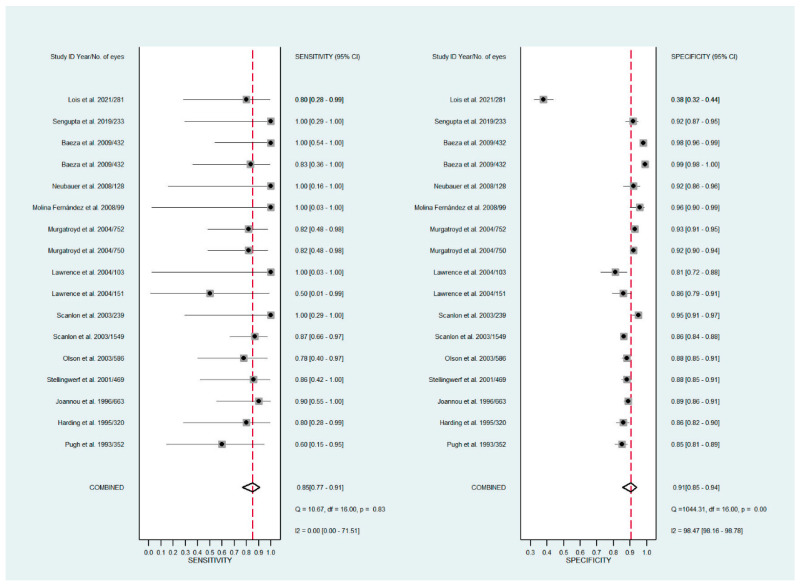
Forest plots for the sensitivity and specificity of mydriatic fundus photography.

**Figure 4:**
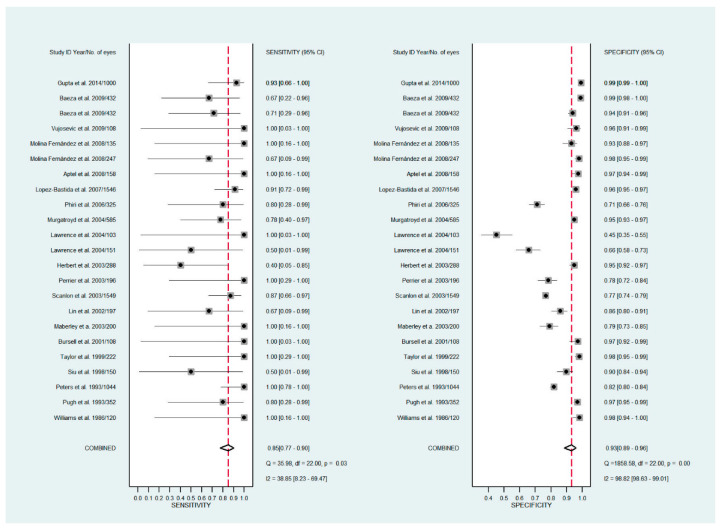
Forest plots for the sensitivity and specificity of non-mydriatic fundus photography.

**Figure 5:**
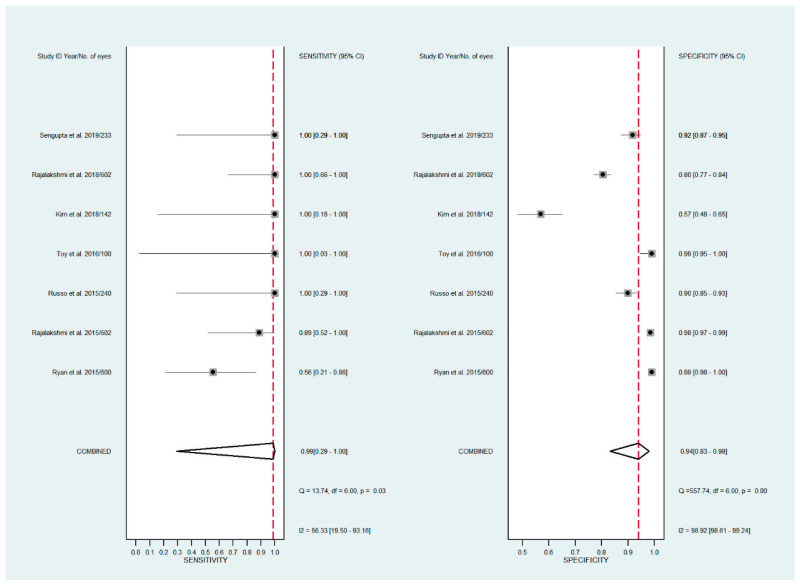
Forest plots for the sensitivity and specificity of smartphone-based fundus imaging.

The pooled sensitivity for all the methods was 0.84 (95% CI: 0.8–0.88) (Figure 2). In terms of sensitivity, mydriatic fundus photography (0.85 [95% CI: 0.77–0.91], I^2^ = 0.0; Figure 3) and non-mydriatic fundus photography (0.85 [95% CI: 0.77–0.9], I^2^ = 38.85; Figure 4) had lower sensitivity than smartphone-based imaging (0.91 [95% CI: 0.85–0.94], I^2^ = 98.47; Figure 5). There was a statistically significant difference between all three groups (*p* < 0.001). Due to the high heterogeneity of smartphone-based imaging studies, the results should be taken with caution.

**Figure 6:**
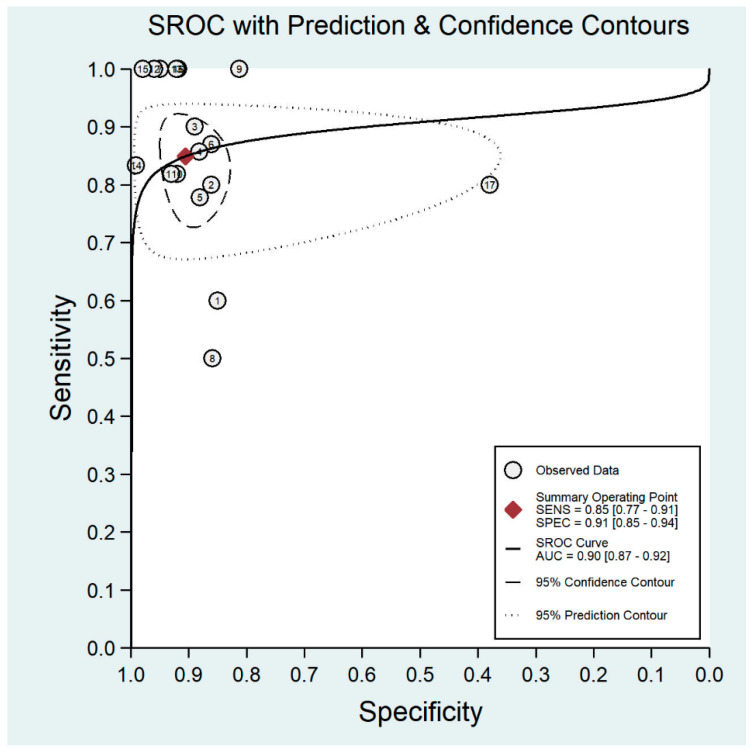
Receiver operating characteristic curve for mydriatic fundus imaging.

**Figure 7:**
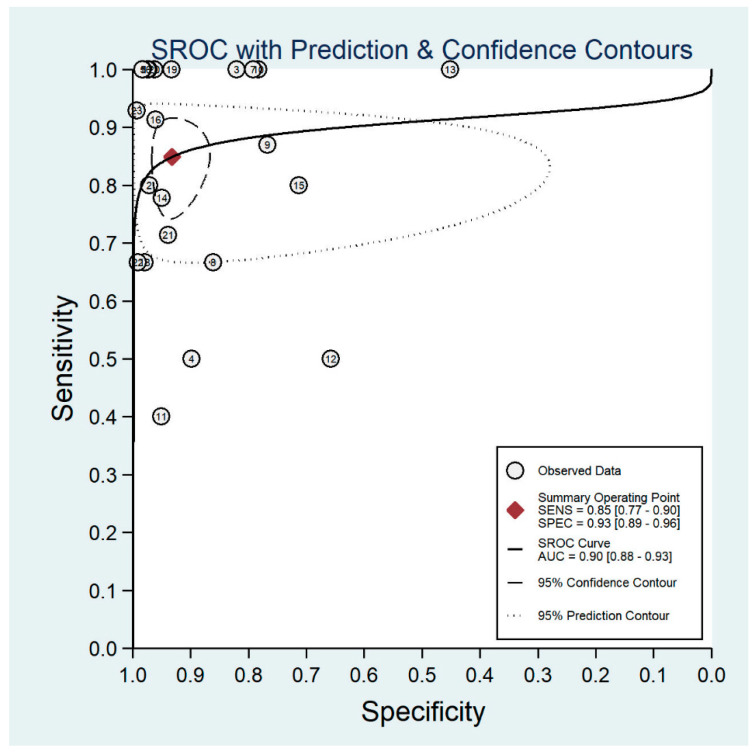
Receiver operating characteristic curve for non-mydriatic fundus imaging.

**Figure 8:**
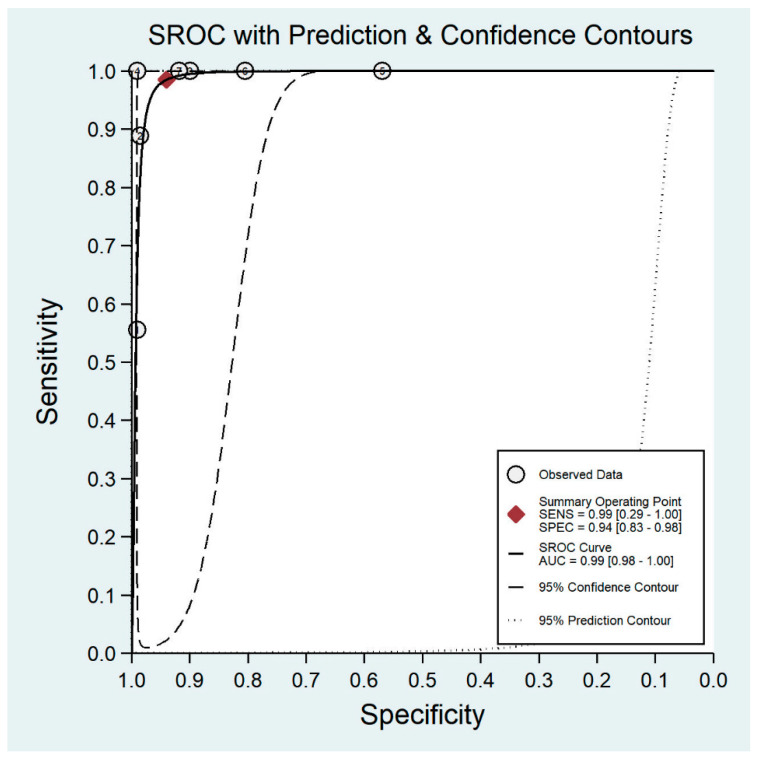
Receiver operating characteristic curve for smartphone-based imaging.

The pooled specificity for all methods was 0.92 (95% CI: 0.89–0.94) (Figure 2). The specificity of mydriatic fundus photography (0.91 [95% CI: 0.84–0.94], I^2^ = 98.47) was not different from that of non-mydriatic fundus photography (0.93 [95% CI: 0.89–0.96], I^2^ = 98.82). The pooled specificity of smartphone-based imaging studies (0.94 [95% CI: 0.83–0.98], I^2^ = 98.92) was significantly better than that of mydriatic (*p* < 0.001) and non-mydriatic fundus photography (*p* < 0.001). There was no difference observed in the specificity for mydriatic and non-mydriatic photography (*p* > 0.05). The receiver operating characteristic (ROC) curves of the analyzed methods are shown in Figure 6, Figure 7 and Figure 8.

The total sample size was the lowest in smartphone-based imaging studies. Moreover, the publication bias factor for smartphone-based imaging was significantly higher than for mydriatic digital fundus photography and non-mydriatic digital fundus photography (b = −8.61, b = −2.59 and b = −7.03, respectively). The pooled sensitivity and specificity for mydriatic methods, i.e., mydriatic fundus photography and smartphone imaging, was 0.85 (95% CI: 0.78–0.90) and 0.92 (95% CI: 0.87–0.95), respectively; it was not different to the sensitivity and specificity of non-mydriatic fundus photography (*p* = 0.827 and *p* = 0.921, respectively).

## 4. Discussion

### 4.1. Fundus Examination vs. Retinal Photography

A DR screening examination could hypothetically include a complete ophthalmic check-up with best-corrected visual acuity after refraction, pupil dilation and state-of-art retinal imaging including wide-field retinal photography with optical coherence tomography [53,54]. This is not performed even in high-resource settings; ideally, a DR screening program should have as few components as possible, be affordable and available, but should ensure appropriate referral [55].

With the increasing prevalence of diabetes, one could consider ophthalmology as under-resourced in some parts of the world. However, even with a sufficient number of ophthalmologists available, employing them to screen every individual with DM is not feasible and likely to be inefficient use of resources [14,56]. As a consequence, in some studies fundoscopy for DR screening was successfully performed by ophthalmological optometrists [20,57,58,59,60], general practitioners [61,62], trained technicians [63] or nurses [64]. Although in a single study consultants performed better than non-consultant staff in grading DR, the variability of opinions was significant even for consultants [65]. In another study, the sensitivity and specificity of slit-lamp examination for DR detection performed by optometrists was 73% and 90%, respectively, compared to the reference slit-lamp biomicroscopy by ophthalmologists with interest in medical retina [20]. In a Norwegian investigation the sensitivity and specificity of optometrists for DR evaluation of 7-field fundus images was 67% (62–72%) and 84% (95% CI: 80–89%), respectively, when compared to reading by ophthalmologists [66]. Only 5% of optometrists met the required standard of at least 80% sensitivity and 95% specificity which was postulated as the ultimate requirement for DR screening programs [66]. Still, these differences might rather be a matter of briefing for specific tasks, than reflect the competence based on the actual educational background. 

Additional criteria should be considered for a screening test — the test should be inexpensive and non-invasive. Screening techniques cannot be expected to perform as well as detailed investigative techniques but should be comparable with the original method [67]. In clinical studies the agreement between ophthalmoscopy and color fundus photography grading by various methods ranges from 34.0% to 86.3% [37]. Interestingly, regarding the grading of DR, there is evidence indicating that color photography is superior to fundoscopy alone [10,20,37,63,65,68,69], and particularly to direct ophthalmoscopy [17,33]. Schachat et al. reported that clinical examination underestimates the prevalence of DR when compared to photography gradings (7.7% vs. 8.7%, respectively) [10]. In another study, the sensitivity and specificity for ophthalmoscopy compared to grading of 7-field fundus photographs for the detection of any DR was 51% and 91%, respectively [70]. Even worse rates of performance were reported in an investigation by Lin et al. where the sensitivity of ophthalmoscopy for DR screening compared with 7-field photography was 34%, with a specificity of 100% [37]. Pugh et al. found that the sensitivity of an ophthalmologist in detecting DR was 33% and it was even worse (sensitivity 14%) for a physician’s assistant when compared to the reference standard, the 7-field photography [16]. Another study reported that ophthalmoscopy missed approximately 50% of eyes with microaneurysms only when compared to fundus photography [71].

It was hypothesized that macular edema with a few hard exudates could be easier to detect in fundoscopy than in non-stereoscopic photography [68]. Nevertheless, such a finding was not confirmed in clinical studies [31,65]. In an investigation by Taylor et al. maculopathy was reported in 147/4312 eyes with camera screening and only in 95/4312 eyes by ophthalmoscopy alone (*p* < 0.001); moreover, ophthalmoscopy underestimated the presence of hard exudates (*p* < 0.001) [65]. A disadvantage of the fundus camera is its cost; however, without such a camera, documenting minimal changes over time might be difficult [63]. However, fundus photography offers the benefit of providing a record of retinopathy which can be used at a later date to document the progression of retinopathy or response to treatment. Currently, it might be difficult to consider eye fundus examinations as a method for DR screening using the resources efficiently.

### 4.2. Monoscopic vs. Stereoscopic Fundus Photography

Both the original Airlie House DR classification used in the Diabetic Retinopathy Study [72,73,74], and the modified DR classification used in the Early Treatment Diabetic Retinopathy Study, employed 7-field stereographic photography [75] to determine the grade of DR. In stereographic retinal photography a stereo image is obtained by taking photographs from two slightly different positions and merging these images enables a perception of depth [76].

The perception of depth in assessing DR severity should help us to determine the presence of macular edema, to differentiate neovascularization from intraretinal microvascular abnormalities, and to detect pre- and intraretinal hemorrhages [77]. Despite the potential benefits, acquisition and grading of stereoscopic images is time-consuming and doubles the number of light flashes that the patient must endure [76]. Moreover, the technique depends on the experience of photographers, as left and right images must be equally sharp and illuminated in each pair [78,79]. For the graders, special equipment such as optical viewers or goggles is needed to achieve the stereo depth and to review them [76]. The perception of stereoscopy is dependent on the observer’s capability to fuse stereoscopically [76]. There is evidence indicating that obtaining stereoscopy is not critical for the assessment of DR severity, and monoscopic photography can equal the reliability of stereo photography for full ETDRS DR severity scale grading [76]. Moreover, it might be questionable whether the cost and logistical concerns involved in obtaining 7-field images either conventionally, or digitally, would make the method practical and cost-effective for widespread screening [63,80].

### 4.3. DR Grading

Within the analyzed studies, two thresholds for DR detection were most commonly used: VTDR or any symptoms DR. VTDR is usually defined as severe non-proliferative, proliferative retinopathy and/or macular oedema in at least one eye [81]. Treatment for VTDR is agreed upon universally [82]: laser treatment is effective [83,84], and vascular endothelial growth factor inhibitors (anti-VEGFs) can improve the results of treatment in diabetic maculopathy [85,86] and in some cases of proliferative DR [87,88]. Patients with mild nonproliferative DR (which is indicated by the presence of at least 1 microaneurysm) do not require any ophthalmic treatment. Thus, positive screening of patients with any symptoms of DR could not be considered appropriate. On the other hand, the rate of DR deterioration is reduced by improved control of blood glucose [89,90,91] and blood pressure [92,93], and this could be some benefit of screening patients with any DR.

In terms of methodological correctness and the principles of meta-analysis, future DR screening research should focus solely on the epidemiology of VTDR. One should consider that the lower the prevalence of a specific disease, the greater the meticulousness and usefulness of the meta-analysis performed as regards the investigated diagnostic tests, which are employed in clinical practice. As mentioned previously, the estimated prevalence of any DR among diabetic is significantly higher than the prevalence of VTDR (34.6% vs. 10.2%, respectively) [5]. Also VTDR could be considered as the main outcome of interest of DR screening programmes.

### 4.4. Mydriatic Versus Non-Mydriatic Fundus Photography

Seven-field mydriatic photography is considered as the gold standard for fundus imaging, however, the inconvenience and risks associated with mydriasis must be considered. Even when using a short acting mydriatic (tropicamide), dilating the pupils can cause discomfort, especially for those who plan to return to work after being screened or need to drive a car to reach the screening facility [65]. Moreover, pupil dilation is time-consuming, both for the patient and also for the examiner, thus negatively influencing efficiency. Finally, as the use of such agents is not popular with patients, it might lead to poorer compliance [25,94]. For example, in a study by Natarajan et al. 9.4% of patients did not agree to participate in the screening due to waiting time and potential discomfort associated with pupil dilation [95]. Non-mydriatic imaging is a faster and less expensive option than mydriatic photography [68].

Importantly, diabetes is concerned as a risk factor for presenting with a small pupil [96,97]. The pupillary dysfunction demonstrated in diabetes is related to autonomic neuropathy and primarily involves the sympathetic innervation of the iris dilator [98]. Applying a mydriatic agent could potentially lead to improving the quality of imaging in these cases. However, the loss of sympathetic tonus in individuals with diabetes restricts the utility of commonly used topical anticholinergic agents resulting in inadequate pupil dilation [99]. Sympathetic denervation is correlated with the duration of the disease and the development of systemic autonomic neuropathy [100]. Diabetic patients might respond relatively poorly to mydriasis with topical tropicamide 1%; pupil dilation might be achieved in these patients by additional application of topical phenylephrine [23,96,101].

In a clinical DR screening study by Murgatroyd et al. mydriasis reduced the proportion of ungradable photographs from 26% to 5% (*p* < 0.001) [24]. In another study up to 29.2% of non-mydriatic images were poorly focused, and as a consequence, partly ungradable [68]. In an investigation by Pugh et al. 14% of undilated and 3.7% of dilated images were found ungradable; importantly, after mydriasis most of the ungradable photographs (42/50) became gradable [16]. Similar results were noted by Baeza et al. who reported that 15.3–17.6% of non-mydriatic images but only 1.4–2.1% of the mydriatic images were ungradable [28]. In a study by Peters et al. the rate of ungradable non-mydriatic images was 32%; patients with ungradable images were older (56.0 vs. 46.6 years) and had a pupil size <4 mm (27% vs. 7%) [32]. Pharmacologic dilation might not only enhance the gradability of fundus photographs but also their accuracy [16,43]. After pupil dilation, some retinal findings such as venous beading or nerve fiber layer hemorrhages, are more probable to be detected [25]. Moreover, in a dark iris population e.g. in Indian eyes, non-mydriatic digital imaging might result in an even higher (30.6–31%) rate of poor quality photographs, resulting in low sensitivity and restricting the use of this technique [43]. The diminished sensitivity of non-mydriatic photographs could be acceptable if a greater percentage of patients would agree to complete the screening process [102]; however, such a finding was not confirmed in clinical trials. Pupil dilation might be used when the quality of the obtained images is poor, e.g. in older patients with advanced cataract or senile miosis [25]. Scotland introduced the concept of staged mydriasis into their screening programme, only dilating those patients having poor-quality images without mydriasis [82]. The image quality is assessed by the technician taking the images. Recently, the numbers needing dilation have currently risen to 34% [82]. In a single study by Molina Fernández et al. selective mydriasis, based upon the decision taken by the family doctor who performed the imaging, did not improve the screening performance [26]. Regardless of the type of the device used, the photographs should be taken on the dilated eye, as this significantly improves the sensitivity and decreases the rate of ungradable images. Selective mydriasis did not improve performance of DR screening.

Several of the analyzed studies are more than 10 years old, and one must consider that in recent years there has been a technical development in fundus cameras. First, advancements in the field of optical sources and detectors have led to miniaturization of optical assemblies at a lower cost. In line with these developments, miniature table-top fundus camera system designs have emerged that provide retinal images comparable to those of traditional fundus cameras [103].Camera systems have evolved to boast sharper images, having a higher resolution, pupil tracking, and, most recently, portability. Potentially, an improvement in camera optics could result in decreasing the TFR rate. On the other hand, this has not been proved in clinical trials.

### 4.5. Single vs. Multiple-Field Imaging

One major concern in single-field imaging is that a smaller area of the retina is imaged; particularly the nasal retina is of importance for a valid evaluation of the DR stage [78]. From a mathematical point of view, a 30° angle field-of-view is equal to visualizing the retinal area of 56.4 mm^2^, while a 45° angle equals to the visualization of a 124.8 mm^2^ area [104]. In these terms, a retinal area visualized with a single 45° image cannot be considered equivalent to seven 30° shots; with two- or three-field 45° images the area could be comparable. 

Different protocols were applied with regional DR screening programs, e.g., a single-field 45° photography in Singapore [105], two-field 45° photography in England [106], or five-field 45° photography in France [107]. In the study of Aptel et al. there was a major difference seen in the sensitivity of detecting DR between single-field and three-field 45° non-mydriatic photographs (76.92% vs. 92.31%, respectively; *p* < 0.001) [25]. The study by Perrier et al. presented no significant difference in sensitivity between two, three and four-field non-mydriatic photography (95.7%, 97.6% and 97.6%, respectively) [38]. Moreover, additional images reduced the specificity (which was 78.1%, 71.9% and 65.6% for two-, three- and four-field imaging, respectively) and led to a higher rate of ungradable images (14.2%, 18.3% and 18.3%, respectively; *p* values not stated) [38]. The poor quality of adding extra-field to two-field imaging translated into an increase of 6.2% in the rate of referral to an ophthalmologist [38]. Baeza et al. noted that by increasing the number of fields from one to three the sensitivity slightly increased (from 68% to 79%) [28]. Importantly, applying mydriasis led to a decrease in the rate of ungradable images (from 15.3–18.3% to 1.4–2.1%) [28]. In another study the performance of two-field evaluation was similar to single-field photography; including nasal images did not bring added value to macular images [20]. Moreover, despite mydriasis, the nasal images had poorer quality than macular images (3.5–8.1% of nasal images were ungradable) [20]. These findings are analogous to the meta-analysis by Hu et al. who reported that single-field non-mydriatic photography might be inadequate to detect DR [15].

### 4.6. Handheld and Smartphone-Based Devices

To expand screening programs into rural areas it would be beneficial to have access to low-cost portable, easy to operate, and high image quality fundus cameras [108]. Tran et al. have shown that it is possible to construct a hand-held mydriatic fundus camera prototype at a cost of less than 1000 USD [109]. Their front-end module was retrofitted to go with several consumer cameras; however, those with smaller CMOS (Complementary Metal Oxide Semiconductor) sensors showed loss of image detail or increased image noise compared to larger CMOS devices [109]. In the following years, several portable eye fundus cameras were developed which have a digital camera incorporated. These include the Smartscope Pro (Optomed, Oulu, Finland) commercialized as Pictor (Volk Optical, Mentor, OH, USA), Horus DEC 200 (MiiS, Nsinchu, Taiwan), Genesis-D (Kowa, Nagoya, Japan), Signal (Topcon Corporation, Tokyo, Japan), Dragonfly (Eyefficient; Aurora, OH, USA), VersaCamTM DS-10 (Nidek, Gamagori, Japan) or Visuscout 100 (Carl Zeiss Meditec AG, Jena, Germany).

Another option for retinal imaging is the use of a smartphone’s in-built camera. A smartphone can be used to capture pictures of the posterior segment of the eye during slit-lamp indirect ophthalmoscopy with a 78 D lens [110,111]. Haddock et al. [112] and Bastawrous [113] suggested using the coaxial light source of the phone rather than that of the slit-lamp; in their technique the phone is being kept in one of the examiners hand, while the other hand is holding a 20 D or 28 D lens. For examinations performed in general anesthesia, additionally a Koeppe contact lens was applied, which was useful in receiving a wider field of view, keeping the lids open and the cornea wet [112]. Images obtained with a 20 D lens have a smaller imaging area of <45° when compared to a combination of a 60 D with a 90 mm focal length lens (area of 92°) [114,115]. A special attachment which is designed to hold a specific lens at a prescribed, but adjustable distance from the camera lens, might improve the ease-of-use of such imaging methods [116,117]; and such an attachment can be 3D-printed [116].

Currently, several adapters for cell phones have become commercially available: D-Eye (D-Eye, Padova, Italy), PanOptic + iExaminer (Welch Allyn, Skaneateles Falls, NY, USA), MII RetCam (MII RetCam Inc., Coimbatore, India), iNview/Vistaview (Volk Optical, Mentor, OH, USA) or the Peek Vision (Nesta, London, UK) [29,118]. The PanOptic and D-Eye have limited imaging fields (25° and 20°, respectively). Interestingly, the Fundus-On-Phone System (Remidio, Bengaluru, India) is smartphone based, but not handheld. The technical details and a review of the currently developed systems was published elsewhere [103] and does not fall within the scope of this paper.

A significant limitation of several smartphone-based systems is the requirement of mydriasis. Moreover, it might be difficult to consider the resolution of a smartphone’s in-built camera (particularly in older phones, which have been used in several studies) to that of a professional desktop camera. Another problem is glare, improper exposure or difficulties in capturing ideally sharp images [103,119]. For example, iPhone’s built-in flash has a fairly high intensity, and efforts are made to design imaging systems with an external light source with varying intensity levels [103]. Finally, sophisticated skill is required to perform the imaging as the beam alignment is problematic, and stability of the camera is required [103]. Thus, unless the examiner is already adept at indirect ophthalmoscopy, it can be challenging to obtain high-quality images that are useful for evaluation [120]. Some might prefer to use portable cameras that have slit lamp attachments. On the other hand, a single study has shown that medical students who were previously unfamiliar with indirect ophthalmoscopy were able to successfully acquire images after 15 minutes of training [121], and some of them preferred smartphone ophthalmoscopy compared to conventional direct ophthalmoscopy [122].

### 4.7. Ultrawide-Field Imaging

Ultrawide-field scanning laser ophthalmoscopy (UWF-SLO) employs confocal laser scanning microscopy combined with a concave elliptical mirror, having the capability of capturing up to 200° of the retina in a single image, without pupil dilation in less than one second [123]. With the steering function it is possible to obtain a greater field under mydriasis with a light inside the camera guiding the patients’ eye [79]. During the examination a low-powered green (532 nm) and red light (633 nm) simultaneously scan the retina and choroidal tissue; a composite picture is created by digital combination of the two wavelengths [50]. By scanning a smaller area (100° instead of 200°) it is possible to obtain images having higher resolution up to 11 µm [50]. Although ultrawide images can be obtained with or without mydriasis, a study by Rasmussen et al. showed that the quality of mydriatic and non-mydriatic images obtained with Optos 200Tx (Optos, Dunfermline, United Kingdom) did not differ significantly [79]. One should mention that currently there are a variety of Optos devices enabling UWF-SLO imaging; it is also possible to obtain 102-degree UWF-SLO images with Spectralis (Heidelberg Engineering, Heidelberg, Germany) [124]. 

An advantage of UWF-SLO is assessment of peripheral pathologies which could be overlooked if a smaller angle is imaged [125,126]. It was hypothesized that a subset of DR patients might exhibit peripheral distribution of retinal lesions, unavailable for visualization in fundus photography [50,127]. Moreover, one-third of retinal hemorrhages and/or microaneurysms, intraretinal microvascular abnormalities and new vessels elsewhere might be situated outside the ETDRS fields, and visible in UWF-SLO but not in 7-field ETDRS photography [126]. UWF-SLO has, as well, the potential of identifying peripheral retinal lesions and vitreous pathologic findings [128]. Another potential benefit could be the reduction in the rate of ungradable images due to better imaging technology [127]. In some of the UWF-SLO systems obtaining fluorescein angiography images is possible [129].

A study by Silva et al. showed that UWF-SLO may underdiagnose proliferative DR [50]. This was presumably associated with colour distortion from the machine and, therefore, requires significant magnification of the images to evaluate discrete retinal lesions. The recently released Clarus 500 and Clarus 700 (Carl Zeiss Meditec AG, Jena, Germany) capture “true-color” images that may potentially enable more accurate identification of DR lesions, although this has yet to be demonstrated in clinical trials [130]. Within the currently published studies, images obtained with Clarus were consistent with current UWF-SLO devices in assessing the severity of DR, with no statistically significant difference in patient or technician preference, and image acquisition time [131,132,133]. The Eidon confocal scanner (Centervue, Padova, Italy) couples confocal imaging with natural white-light illumination to obtain a true-colour image using a white LED (440–650 nm). The Spectralis (Heidelberg Engineering, Heidelberg, Germany) has a dedicated Spectralis MultiColor Module, which is not available in the standard version of the device and uses three laser wavelengths simultaneously to receive color images; thus, the basic version of device cannot be considered as optimized for DR screening. Potentially, UWF-SLO could be less susceptible to media opacities or decreased pupil diameter compared with conventional fundus photography [78]. However, in another study, all images of patients with proliferative DR were found ungradable due to glare associated with media opacities in a dense cataract or vitreous hemorrhage [51]. In the investigation by Aiello et al., UWF-SLO imaging in a clinical setting increased the frequency of DR identification nearly two-fold but the agreement with ETDRS 7-field imaging was moderate [134].

One disadvantage of the UWF-SLO technology compared to other approaches is that it is still more costly [78]. This issue could be critical to wide-spread use of UWF-SLO for DR screening, as a screening examination should be inexpensive. For example, expenditures on the English DR Screening Program which employed fundus cameras were approximately 85.6 million USD or 40 USD per person screened [106]. With UWF-SLO devices, which are significantly more expensive than fundus cameras, these costs could even be higher. Although the results of the English program are excellent, high costs preclude implementation of this strategy worldwide; in several studies emphasis is placed on new, cost-effective systems. On the other hand, Lois et al. showed that savings associated with UWF-SLO for DR assessments are greater than for 7-field photography mainly due to longer time to obtain and read images in the 7-field photography technique [30]. Future research may aim to clarify the association of peripheral diabetic lesions with the stage of DR [135]. One might discuss whether UWF-SLO is advisable for screening of high-risk DR or proliferative DR [136]. This aspect requires further validation [51]. 

### 4.8. Multimodal Imaging Techniques and Potential Future Directions

Multimodal imaging techniques employ several imaging methods to examine a particular finding. Quantitation of retinal thickness and precise topographic mapping of the retina have been useful in assessing retinal thickness in both non-clinically significant macular edema and clinically significant macular edema [137]. Optical coherence tomography (OCT) is more reproducible and more sensitive to follow changes in retinal thickness when compared to fundus photography [138]. Technically, it is possible to obtain simultaneous or immediately sequential fundus photographs and OCT images [139]. Such devices are commercially available, e.g. in the Maestro2 (Topcon Corporation, Tokyo, Japan) or the Revo FC (Optopol Technology Sp. z o.o., Zawiercie, Poland) [140]. Importantly, adding OCT to the assessment of maculopathy improves the sensitivity and specificity of detecting clinically significant macular edema as well as any maculopathy (i.e., exudates only) [52]. Both of the aforementioned devices also allow obtaining OCT-angiography images. Nevertheless, current limitations of OCT angiography include a small field of view, projection and motion artifact, and inability to assess flow and filling speeds or vascular competence by assessing dye leakage [141]. OCT can also be combined with UWF-SLO imaging [52].

Other technical advantages may play a role in multimodal DR assessment in the future [141]. Enhanced depth imaging OCT or swept-source OCT could allow improved choroidal visualization [142]. Choroidal thickness was shown to be altered in patients with diabetes and diabetic choroidopathy; it was suggested that a change in choroidal thickness may precede any retinopathy [143,144,145]. Adaptive optics allow a noninvasive acquisition of images of the retina with cellular-level resolution and assessment of individual photoreceptor cells [146]. Hyperspectral imaging might be a promising way to measure oxygenation in the retinal blood vessels; this is important because hyperglycaemia is known to increase retinal oxygen consumption [147,148].

## Figures and Tables

**Table 1:** Testing accuracy in studies on imaging modalities used for detecting diabetic retinopathy.

Technique	Study	Number of Eyes	Imaging Details and Device	Pupil Dilation	Technical Failure Rate [%]	Reference Standard, (Testing Accuracy Analyzed for)	Sensitivity [%] (95% CI) *	Specificity [%] (95% CI) *	Kappa (95% CI)
Mydriatic digital fundus photography	Pugh et al. 1993 [16]	352	3-field 45° (Canon CR3)	Y	3.7	7-field 30° Zeiss, any DR	61	85	0.74 (0.66–0.82)
	Harding et al. 1995 [17]	320	3-field 45° (Canon CR4-45NM)	Y	1.8	Slit-lamp biomicroscopy, VTDR	89 (80–98)	86 (82–90)	
	Joannou et al. 1996 [18]	663	60° photography (Canon CF-60)	Y	N/A	Dilated ophthalmoscopy, any DR	93	89	
	Stellingwerf et al. 2001 [19]	469	2-field 50° (Canon CF-60)	Y	2	7-field 30°, any DR	83	88	0.71
	Stellingwerf et al. 2001 [19]	469	2-field 50° (Canon CF-60)	Y	2	7-field 30°, VTDR	95	99	0.71
	Olson et al. 2003 [20]	586	2-field 50° (digital Topcon camera, manual assessment)	Y	4.4	Dilated ophthalmoscopy, any DR	83 (77–89)	79 (75–83)	
	Olson et al. 2003 [20]	586	1-field 50° (digital Topcon camera, manual assessment)	Y	4.4	Dilated ophthalmoscopy, any DR	80 (74–86)	88 (84–91)	
	Scanlon et al. 2003 [21]	1549	2-field 45° (Topcon NRW5S)	Y	3.7	Slit-lamp biomicroscopy, VTDR	87.8	86.1	0.67–0.75
	Scanlon et al. 2003 [22]	239	2-field 45° (Canon CR6)	Y	1.5	7-field 30°, referrable DR	87.4 (83.5–91.5)	94.9 (91.5–98.3)	0.8
	Lawrence et al. 2004 [23]	151	3-field 45° (Topcon TRC-NW5SF)	Y	N/A	7-field 30°, any DR	66	86	
	Lawrence et al. 2004 [23]	103	3-field 45° (Topcon TRC-NW6S)	Y	N/A	7-field 30°, any DR	85	81	
	Murgatroyd et al. 2004 [24]	750	1-field 45° (Topcon TRC-NW6S)	Y	7	Slit-lamp ophthalmoscopy, “Referrable DR”	81 (76–87)	92 (90–94)	0.86
	Murgatroyd et al. 2004 [24]	752	3-field 45° (Topcon TRC-NW6S)	Y	6.5	Slit-lamp ophthalmoscopy “Referrable DR”	83 (78–88)	93 (91–96)	0.88
	Aptel et al. 2008 [25]	158	1-field 45°	Y	N/A	Indirect ophthalmoscopy, any DR	89.74	98.3	0.9
	Aptel et al. 2008 [25]	158	3-field 45°	Y	N/A	Indirect ophthalmoscopy, any DR	97.44	98.3	0.95
	Molina Fernández et al. 2008 [26]	99	3-field 45° (Topcon TRC-50 EX)	Y	17.2	Ophthalmological Examination, referrable DR	85 (62.1–96.8)	96.4 (85.1–98.9)	
	Neubauer et al. 2008 [27]	128	1-field 45° (Zeiss Visucam^PRO NM^)	Y	N/A	7-field 30° Zeiss FF450^plus^ images, ETDRS level 35	99 (94–100)	92 (73–99)	0.87 (0.81–0.92)
	Baeza et al. 2009 [28]	432	1-field 45° (Topcon CRW6S)	Y	1.4	7-field 30°, any DR	77 (71–83)	98 (96–99)	0.77
	Baeza et al. 2009 [28]	432	2-field 45° (Topcon CRW6S)	Y	1.6	7-field 30°, any DR	86 (81–91)	95 (92–98)	0.82
	Baeza et al. 2009 [28]	432	3-field 45° (Topcon CRW6S)	Y	2.1	7-field 30°, any DR	85 (80–90)	94 (91–97)	0.81
	Baeza et al. 2009 [28]	432	1-field 45° (Topcon CRW6S)	Y	1.4	7-field 30°, VTDR	82 (72–92)	99 (97–100)	0.84
	Baeza et al. 2009 [28]	432	2-field 45° (Topcon CRW6S)	Y	1.6	7-field 30°, VTDR	95 (89–100)	98 (97–100)	0.91
	Baeza et al. 2009 [28]	432	3-field 45° (Topcon CRW6S)	Y	2.1	7-field 30°, VTDR	95 (89–100)	98 (96–99)	0.89
	Sengupta et al. 2019 [29]	233	3-field 45 degree images (Topcon TRC-50DX)	N	2.6–4.3	Dilated fundus examination, any DR	(92.6–94.9)	(85.5–98.2)	0.68 (0.67–0.73)
	Lois et al. 2021 [30]	281	7-field imaging (Not specified)		6.0	Clinical examination, proliferative DR	85 (77–91)	38 (41–56)	
Non-mydriatic digital fundus photography	Williams et al. 1986 [31]	120	1-field 45° (Kowa or Canon CR3 camera)	N	N/A (excluded)	Dilated fundus examination, any DR	96	98	N/A
	Pugh et al. 1993 [16]	352	1-field 45° (Canon CR3)	N	14	7-field 30° Zeiss, any DR	81	97	0.62 (0.54-0.70)
	Peters et al. 1993 [32]	1044	1-field 45° (Canon CR4)	N	32	Ophthalmological exam, VTDR	100	82	N/A
	Siu et al. 1998 [33]	150	1-field 45° (Canon CR-45UAF)	N	N/A	Indirect ophthalmoscopy, any DR	64 (43–85)	90 (84–96)	
	Taylor et al. 1999 [34]	222	1-field 45° (Canon CR5)	N	N/A	7-field 30° Zeiss, any DR	74 (68–80)	96 (94–98)	N/A
	Taylor et al. 1999 [34]	222	1-field 45° (Canon CR5 Digital)	N	N/A	7-field 30° Zeiss, VTDR	85 (80–90)	98 (96–100)	N/A
	Bursell et al. 2001 [35]	108	3-field 45°, Joslin Vision Network Technology protocol (Topcon TRC NW-5S)	N	2.8	7-field 30° Zeiss FF4 camera, any DR	89	97	0.87
	Maberley et a. 2003 [36]	200	1-field 45° (Topcon TRC NW5SF)	N	1.0	Slit-lamp ophthalmoscopy, any DR	84.4 (79–90)	79.2 (74.1–84.3)	0.85 (0.78–0.92)
	Lin et al. 2002 [37]	197 patients	2-field, 640 × 480 px black-and-white images (Canon CR5-45NM)	N	8.1	7-field 30° Zeiss FF4 camera, referrable DR	78	86	0.4
	Scanlon et al. 2003 [21]	1549	1-field 45° Topcon NRW5S)	N	19.7	Slit-lamp biomicroscopy, VTDR	86.0	76.7	0.67–0.75
	Perrier et al. 2003 [38]	196	2-field 45° (Topcon CRW6)	N	14.2	7-field 30°, any DR	95.7	78.1	0.76
	Perrier et al. 2003 [38]	196	3-field 45° (Topcon CRW6)	N	18.4	7-field 30°, any DR	97.6	71.9	0.71
	Perrier et al. 2003 [38]	196	4-field 45° (Topcon CRW6)	N	18.4	7-field 30°, any DR	97.6	65.6	0.65
	Herbert et al. 2003 [39]	288	1-field 45° (Topcon TRC-NW5S)	N	4	Slit-lamp ophthalmoscopy, any DR)	38.0	95.0	0.84
	Lawrence et al. 2004 [23]	151	1-field 45° (Topcon TRC-NW5SF)	N	N/A	7-field 30°, any DR	66	66	
	Lawrence et al. 2004 [23]	103	1-field 45° (Topcon TRC-NW6S)	N	N/A	7-field 30°, any DR	76	45	
	Murgatroyd et al. 2004 [24]	585	1-field 45° (Topcon TRC-NW6S)	N	36	Slit-lamp ophthalmoscopy (referrable DR)	77 (71–84)	95 (93–97)	1.0
	Phiri et al. 2006 [40]	325	1-field 45° (digital Canon CR6)	N	14.0	7-field 30°, referrable DR	84.1 (65.5–93.7)	71.2 (58.1–81.1)	0.65
	Lopez-Bastida et al. 2007 [41]	1546	1-field 45° (Topcon TRC-NW6S)	N	7.2 (required pupil dilation)	Slit-lamp ophthalmoscopy (VTDR)	100	100	1
	Lopez-Bastida et al. 2007 [41]	1546	1-field 45° (Topcon TRC-NW6S)	N	7.2 (required pupil dilation)	Slit-lamp ophthalmoscopy (any DR)	92 (90–94)	96 (95–98)	0.89
	Aptel et al. 2008 [25]	158	1-field 45° (Topcon TRC-NW6S)	N	11.4	Indirect ophthalmoscopy, any DR	76.9	99.2	0.82
	Aptel et al. 2008 [25]	158	3-field 45° (Topcon TRC-NW6S)	N	13.3	Indirect ophthalmoscopy, any DR	92.3	97.5	0.9
	Molina Fernández et al. 2008 [26]	247	3-field 45° (Topcon TRC-50 EX)	N	38.4	Ophthalmological Examination, referrable DR	66.7 (41–86.7%)	98 (89.1–99.9)	
	Molina Fernández et al. 2008 [26]	135	3-field 45° (Topcon TRC-50 EX)	N (not routine, in selected cases)	27.4	Ophthalmological Examination, referrable DR	76.9 (56.4–91)	93.4 (84.1–99.2)	
	Vujosevic et al. 2009 [42]	108	3-field, 1392 × 1040 px (Nidek)	N	N/A	7-field 30° Topcon TRC 50IA, referrable DR	82	92	0.74 (0.61–0.87)
	Vujosevic et al. 2009 [42]	108	1-field, 1392 × 1040 px (Nidek)	N	N/A	7-field 30° Topcon TRC 50IA, referrable DR	71	96	0.67 (0.5– 0.80)
	Baeza et al. 2009 [28]	432	1-field 45° (Topcon CRW6S)	Y	15.3	7-field 30°, any DR	68 (60–75)	98 (96–100)	0.68
	Baeza et al. 2009 [28]	432	2-field 45° (Topcon CRW6S)	Y	17.1	7-field 30°, any DR	76 (70–83)	94 (90–98)	0.77
	Baeza et al. 2009 [28]	432	3-field 45° (Topcon CRW6S)	Y	17.6	7-field 30°, any DR	79 (73–86)	94 (90–98)	0.77
	Baeza et al. 2009 [28]	432	1-field 45° (Topcon CRW6S)	Y	15.3	7-field 30°, VTDR	67 (54–80)	99 (98–100)	0.75
	Baeza et al. 2009 [28]	432	2-field 45° (Topcon CRW6S)	Y	17.1	7-field 30°, VTDR	80 (69–91)	99 (98–100)	0.85
	Baeza et al. 2009 [28]	432	3-field 45° (Topcon CRW6S)	Y	17.6	7-field 30°, VTDR	82 (81–92)	99 (98–100)	0.86
	Gupta et al. 2014 [43]	1000	3-field, Zeiss Visupac 450+	N	25.6	Dilated fundoscopy, VTDR	91.1	99.3	0.92
Smartphone-based imaging	Ryan et al. 2015 [44]	600	iPhone 5 + 20D lens	N	1.5	7-field dilated fundus photography, any DR	81 (75–86)	94 (92–96)	0.76 (0.71–0.82)
	Ryan et al. 2015 [44]	600	iPhone 5 + 20D lens	N	1.5	7-field dilated fundus photography, VTDR	54 (40–67)	99 (98–100)	0.64 (0.52–0.76)
	Rajalakshmi et al. 2015 [45]	602	Android Phone + Remidio Fundus on Phone imaging system (4-field)	Y	N/A	7-field dilated fundus photography, any DR	92.7 (87.8–96.1)	98.4 (94.3–99.8)	0.90 (0.85–0.95)
	Rajalakshmi et al. 2015 [45]	602	Android Phone + Remidio Fundus on Phone imaging system (4-field)	Y	N/A	7-field dilated fundus photography, VTDR	87.9 (83.2–92.9)	94.9 (89.7–98.2)	0.80 (0.71–0.89)
	Russo et al. 2015 [46]	240	iPhone 5 + D-Eye Adapter (5-field)	Y	3.7	Slit-lamp biomicroscopy, no apparent DR	96 (90–98)	90 (83–95)	0.78 (0.71–0.84)
	Ryan et al. 2015 [44]	600	iPhone 5 + 20 D lens	Y	1.8	7-field dilated fundus photography, any DR	50 (43–56)	94 (92–97)	0.48 (0.41–0.56)
	Ryan et al. 2015 [44]	600	iPhone 5 + 20 D lens	Y	1.8	7-field dilated fundus photography, VTDR	59 (46–72)	100 (99–100)	0.71 (0.6–0.82)
	Toy et al. 2016 [47]	100	iPhone 5s + Volk ClearField lens + Paxos Scope adapter	Y	4.0	Dilated fundus examination, referrable DR	91	99	0.7
	Kim et al. 2018 [48]	142	iPhone 5S + Cellscope Retina optical system (5-field)	Y	16	Dilated fundus examination, referrable DR	93.3	56.8	0.55–0.63
	Rajalakshmi et al. 2018 [49]	602	Android Phone + Remidio Fundus on Phone imaging system (4-field) + EyeArt AI Algorithm	Y	N/A	Dilated fundus examination, any DR	95.8 (92.9–98.7)	80.2 (72.6–87.8)	0.78 (0.71–0.86)
	Rajalakshmi et al. 2018 [49]	602	Android Phone + Remidio Fundus on Phone imaging system (4-field)+ EyeArt AI Algorithm	Y	N/A	Dilated fundus examination, VTDR	99.1 (95.1–99.9)	80.4 (73.9–85.9)	0.75 (0.67–0.83)
	Sengupta et al. 2019 [29]	233	HTC One M8 + Remidio Fundus on Phone imaging (3-field 45°)	Y	1.7–2.1	Dilated fundus examination, any DR	93.1 (88.3–96.4) 94.3 (89.7–97.2)	89.1 (68.2–92.2) 94.5 (84.9–98.9)	0.55 (0.50–0.57)
Ultrawide-field imaging	Silva et al. 2012 [50]	206	Stereoscopic 100° and 200° images (Optos Resmax)	Y	0.5	7-field dilated fundus photography, any DR	(95–100)	(81–100)	0.95 ± 0.03
	Szeto et al. 2019 [51]	322	Non-stereoscopic 200° (Optos Daytona)	N	7.1	Dilated fundus examination, any DR	67.7 (60.0–74.8)	97.8 (93.6–95.5)	0.63
	Szeto et al. 2019 [51]	322	Non-stereoscopic 200° (Optos Daytona)	N	7.1	Dilated fundus examination, VTDR	72.6 (58.2–84.1)	97.8 (92.7–98.1)	0.71
	Manjunath et al. 2015 [52]	2046	Non-stereoscopic 200° (Optomap P2000)	Y	1.1	Clinical examination, VTDR	84.0 (81–87)	69.0 (67–72)	0.75
	Lois et al. 2021 [30]	281	Optos System (Not specified)		5.0	Clinical examination, proliferative DR	83 (75–89)	54 (46–61)	

* If analyzed for two or more observers, values for each observer or preferably the average for the observers is presented. Abbreviations: CI—confidence interval, DR—diabetic retinopathy, VTDR—vision-threatening diabetic retinopathy.

## Appendix A. Search Strategy

Literature searches of the PubMed and Web of Science databases were conducted in 30 June 2021; the search strategies are as follows. Specific limited update searches were conducted after 30 June 2021. Reference lists of the included studies were also considered as a source of publications.

### Appendix A.1. PubMed Search (Publication Date 1/10/11–06/30/2021)

((“diabetes”[Title]) OR (“diabetic”[Title])) AND ((“retinopathy”[Title]) OR (“macular edema”[Title]) OR (“macular oedema”[Title])) AND ((“screening”[Title]) OR (“imaging”[Title]) OR (“fundus”[Title]) OR (“photography”[Title]) OR (“scanning laser ophthalmoscopy”[Title])). 1339 references.

### Appendix A.2. Web of Science Search (Publication Date 1/10/11–6/30/2021)

(TI=(“diabetes”) OR TI=(“diabetic”)) AND (TI=(“retinopathy”) OR TI=(“macular edema”)) AND (TI=(“screening”) OR TI=(“imaging”) OR TI=(“fundus”) OR TI=(“photography”) OR TI=(“scanning laser ophthalmoscopy”)) Indexes=SCI-EXPANDED, SSCI, A&HCI, CPCI-S, CPCI-SSH, BKCI-S, BKCI-SSH, ESCI, CCR-EXPANDED, IC Timespan=All years. 2051 references.
